# Non-coding RNAs in lung cancer: molecular mechanisms and clinical applications

**DOI:** 10.3389/fonc.2023.1256537

**Published:** 2023-09-08

**Authors:** Ying Liu, Wei Ding, Jianxun Wang, Xiang Ao, Junqiang Xue

**Affiliations:** ^1^ The Affiliated Hospital of Qingdao University, Qingdao, Shandong, China; ^2^ Institute for Translational Medicine, The Affiliated Hospital of Qingdao University, Qingdao Medical College, Qingdao University, Qingdao, Shandong, China; ^3^ School of Basic Medicine, Qingdao University, Qingdao, Shandong, China; ^4^ Department of Rehabilitation Medicine, the Affiliated Hospital of Qingdao University, Qingdao, Shandong, China

**Keywords:** lung cancer, microRNAs, long non-coding RNAs, circular RNAs, biomarker, therapeutic target

## Abstract

Lung cancer (LC) is a heterogeneous disease with high malignant degree, rapid growth, and early metastasis. The clinical outcomes of LC patients are generally poor due to the insufficient elucidation of pathological mechanisms, low efficiency of detection and assessment methods, and lack of individualized therapeutic strategies. Non-coding RNAs (ncRNAs), including microRNA (miRNA), long non-coding RNA (lncRNA), and circular RNA (circRNA), are endogenous regulators that are widely involved in the modulation of almost all aspects of life activities, from organogenesis and aging to immunity and cancer. They commonly play vital roles in various biological processes by regulating gene expression via their interactions with DNA, RNA, or protein. An increasing amount of studies have demonstrated that ncRNAs are closely correlated with the initiation and development of LC. Their dysregulation promotes the progression of LC via distinct mechanisms, such as influencing protein activity, activating oncogenic signaling pathways, or altering specific gene expression. Furthermore, some ncRNAs present certain clinical values as biomarker candidates and therapeutic targets for LC patients. A complete understanding of their mechanisms in LC progression may be highly beneficial to developing ncRNA-based therapeutics for LC patients. This review mainly focuses on the intricate mechanisms of miRNA, lncRNA, and circRNA involved in LC progression and discuss their underlying applications in LC treatment.

## Introduction

Lung cancer (LC) is considered a major obstacle to increasing life expectancy worldwide (1). Globally, LC cases and deaths are rising rapidly. In 2020, GLOBOCAN estimated more than 2.2 million new LC cases occurred (2). LC has become a serious global health concern, bringing significant pain and economic burdens to patients and their families. According to pathological characteristics, LC is mainly classified into two subtypes: small cell lung cancer (SCLC) and non-small cell lung cancer (NSCLC) (3, 4). The clinical outcomes of LC patients are commonly poor due to their unobvious early symptom, lack of efficient prognostic evaluation method, and insufficient understanding of pathogenesis (5, 6). Therefore, elucidating the regulatory mechanisms of LC progression may greatly benefit patients in the adjustment of therapeutic strategies and the identification of valuable biomarkers or targets.

Non-coding RNAs (ncRNAs) are functional RNA transcripts that have no protein-coding capacity (7–9). According to their biological functions, ncRNAs are mainly grouped into housekeeping ncRNA and regulatory ncRNA (10). Housekeeping ncRNAs (e.g., ribosomal RNA) are stably expressed in eukaryotic cells. Their products are essential for maintaining the basic life activity of cells (11). Regulatory ncRNAs are key players in almost all biological processes (12). Based on their structural features, ncRNAs are further categorized into microRNA (miRNA) (13), long non-coding RNA (lncRNA) (14), circular RNA (circRNA) (15), small interfering RNA (16), and PIWI-interacting RNA (17). They participate in the regulation of various biological processes, including transcription, development, and immunity, by altering specific gene expression (18–20). Therefore, their dysregulation is closely correlated with various diseases, such as brain disease, diabetes, and cancer (21–27) NcRNA dysregulation has been reported to contribute to almost all aspects of LC development, including apoptosis, cell cycle, metastasis, and autophagy, as well as cell stemness (28–31). However, investigations of LC-related ncRNAs are still lacking.

In this review, we mainly present the modes of action of miRNA, lncRNA, and circRNA and their regulatory mechanisms involved in the initiation and development of LC. We also explore the underlying utilization of these ncRNAs in LC clinical treatment.

## Types of ncRNA

NcRNAs (e.g., miRNAs, lncRNAs, and circRNAs) are essential regulators in various physiological and pathological processes, such as regeneration, development, immunopathogenesis, intracerebral hemorrhage, and LC (32–36).

### MiRNA

MiRNAs are well-studied small ncRNAs, with a single-stranded structure of 19–25 nucleotides (37). Approximately 2300 miRNAs are found in human cells, and they can serve as post-transcriptional regulators to modulate over 60% of the protein-coding genes (38, 39). The canonical function of miRNAs is to regulate specific gene expression by influencing messenger RNA (mRNA) stability (40). In general, miRNAs suppress gene expression by directly interacting with partially complementary sequences in their target mRNAs (41). The method of gene inhibition relies on the complementary extent between miRNA and target mRNA. Exact matching commonly results in mRNA degradation, whereas partial matching induces translational suppression (42). Moreover, miRNAs recognize and mediate mRNA degradation and/or translational inhibition by recruiting the miRNA-induced silencing complex consisting of Argonaute proteins and GW182 (43). The near-seed or non-seed regions of miRNAs are also required for miRNA-mediated modulation of gene expression (44). In addition, nuclear miRNAs are found to mediate the silencing or activation of transcriptional genes (45–47).

### LncRNA

LncRNAs are the largest type of ncRNAs and comprise 81.8% of the total ncRNAs (48, 49). They exhibit highly specific lineage, spatiotemporal, and tissue/cell-dependent patterns, but their abundance, stability, and conservation are less than mRNA (50, 51). LncRNAs are essential modulators that participate in almost every step of gene expression (52, 53). Their canonical mechanism of action is to inhibit target gene expression by binding to miRNA and imposing an additional post-transcriptional regulation level. LncRNAs can also induce transcription factors (TFs) away from chromatin by serving as molecular sinks, thereby altering gene expression (54). Furthermore, some studies suggest that they function as scaffolds to form scaffolding complexes with effectors, resulting in the alteration of gene expression (55). LncRNAs can also guide the ribonucleoprotein complex to the promoters of downstream target genes, thereby altering the transcriptional activity of genes (56). In addition, lncRNAs also alter gene expression through influencing mRNA processing, maturation, and stability (53).

### CircRNA

CircRNAs are single-stranded ncRNA molecules generated from the pre-mRNA back-splicing process and possess a covalently closed-loop structure (57). The closed ring structure can protect circRNAs from exonuclease-mediated degradation, resulting in their stable existence in various subcellular structures (58). CircRNAs are key modulators in many biological processes, including gene transcription, protein translation, immune response, and carcinogenesis, as well as chemoresistance (59–61). The most widely investigated role of circRNAs is to weaken their effect on target mRNAs by serving as miRNA sponges, ultimately resulting in the alteration of correlated gene expression. These circRNAs commonly possess multiple miRNA response elements (62, 63). CircRNAs also participate in biological processes by influencing the functions of proteins (64–66). Furthermore, EIciRNAs are found to facilitate the RNA polymerase II-mediated transcription of their parental genes by binding to U1 small nuclear ribonucleoproteins (33). CircURI1 regulates the AS of multiple migration-related genes by directly interacting with hnRNPM, leading to the inhibition of gastric cancer metastasis (67). In addition, a small part of endogenous circRNAs, which contain open reading frames, have been shown to translate into peptides or proteins (68). However, their potential functions are still unclear.

## NcRNA expression in LC

Differentially expressed ncRNAs play crucial roles in LC occurrence and development (69). Zhang et al. revealed 190 differentially expressed miRNAs between pleural effusion induced by lung adenocarcinoma (LUAD) and pleural effusion induced by tuberculosis, including 99 highly expressed miRNAs and 91 low expression miRNAs. These miRNAs probably influenced the production of pleural effusion via tumor immune response (70). In another study, Zeng et al. distinguished 24 aberrantly expressed miRNAs between NSCLC patients with tumor shrinkage of ≤30% after radiotherapy and patients with tumor shrinkage of 30%–50%, 11 (6 upregulated and 5 downregulated) between patients with tumor shrinkage of ≤30% and patients with tumor shrinkage of ≥50%, and 35 between patients with tumor shrinkage of 30%–50% and patients with tumor shrinkage of ≥50% (71). Furthermore, by comparing the plasma of LUAD patients with benign pulmonary nodule patients, Tong et al. confirmed 1762 differentially expressed lncRNAs in LUAD patients, 946 in lung squamous cell carcinoma patients, and 298 in SCLC patients (72). Huang et al. revealed 177 highly expressed lncRNAs and 215 low expression lncRNAs in the exosomes of LUAD pleural effusion compared with that of benign pleural effusion (73). In addition, Cai et al. performed high-throughput sequencing and identified 598 differentially expressed circRNAs between LUAD patients with bone metastasis and patients without bone metastasis, among which 238 were upregulated and 360 were downregulated (74).

## NcRNA and cancer-related pathways in LC

**Figure 1:**
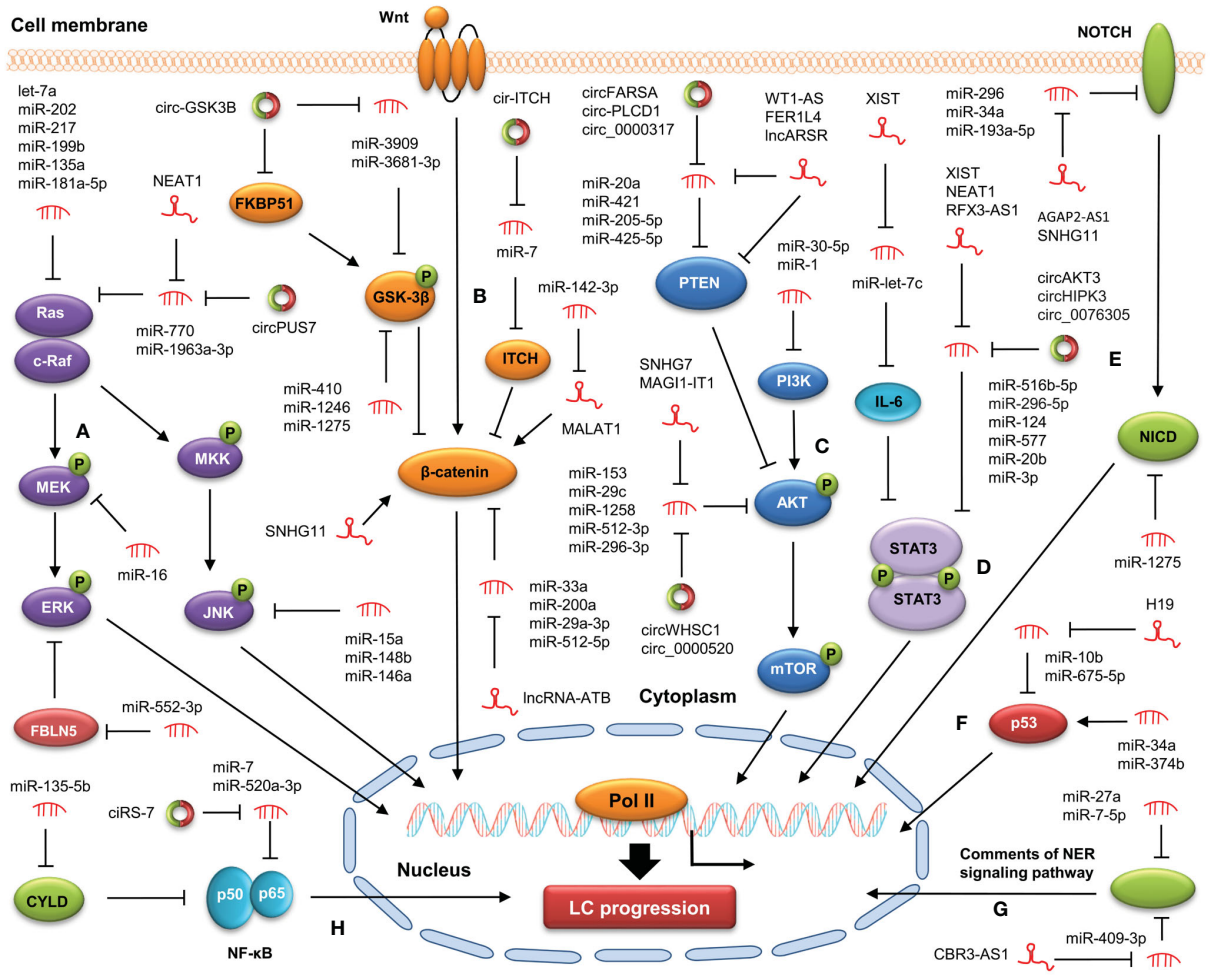
Modulation of ncRNAs on cancer-related signaling pathways in LC. NcRNAs are involved in LC progression by targeting the MAPK **(A)**, WNT/β-catenin **(B)**, PI3K/AKT **(C)**, STAT3 **(D)**, Notch **(E)**, p53 **(F)**, NER **(G)**, NF-κB **(H)** signaling pathways.

Recent studies suggest that the crosstalk between ncRNA and oncogenic signaling pathway is involved in LC initiation and development (75–77) (**Figure 1**). A better understanding of ncRNA action in targeting cancer-related signaling pathways may be of great benefit to the prevention and treatment of LC.

### PI3K/AKT pathway

The PI3K/AKT signaling pathway is a conserved signaling cascade involved in various biological processes, including growth, differentiation, metabolism, and survival. The aberrant activation of this pathway contributes to LC progression (78). MiRNAs play vital roles in LC progression by targeting the PI3K/AKT signaling pathway. For example, in a study by Niu et al., miRNA let-7c-3p was found to inactivate the PI3K/AKT signaling pathway through downregulating PIK3CA, thereby suppressing proliferation and migration in NSCLC cell lines H460 and A549 (79). Furthermore, Shi et al. showed that miR-514b-5p facilitated NSCLC progression by downregulating SGTB and enhancing the PI3K/AKT signaling pathway (80). LncRNAs and circRNAs are also key regulators of the PI3K/AKT signaling pathway during LC progression. For example, high expression of LASTR was observed in both LUAD and lung squamous cell carcinoma (LUSC) samples. LASTR overexpression increased the levels of transforming growth factor alpha by sponging miR-137, thereby activating the PI3K/AKT signaling pathway and ultimately leading to the facilitation of LC progression (81). Liu et al. demonstrated that circGRAMD1B enhanced the activity of the PI3K/AKT pathway by increasing SOX4 levels via sequestering miR-4428, resulting in the facilitation of LUAD progression (82).

### MAPK pathway

The MAPK signaling pathway is a highly conserved pathway that plays an important role in maintaining cellular behaviors and processes, including epithelial-to-mesenchymal transition (EMT), apoptosis, and migration (83). NcRNA dysregulation has been demonstrated to participate in LC progression by modulating the MAPK signaling pathway. For example, exosomal miRNA let-7c-5p and miR-181b-5p was found to repress EMT in bronchial epithelial cells (BEAS-2B) by suppressing the MAPK signaling pathway, thereby inhibiting the invasion of BEAS-2B cells (84). Besides, Shi et al. showed that miRNA let-7a overexpression significantly suppressed the activity of the MAPK signaling pathway by downregulating Ras, p-Raf1/Raf1, and p-MEK1/MEK1 via targeting Rsf-1 in LC cells, resulting in the repression of cell proliferation after radiotherapy (85). Furthermore, Zhu et al. demonstrated that the levels of LINC00649 expression were remarkably increased in LUSC cells, and its upregulation facilitated the occurrence and development of LUSC. Mechanistically, LINC00649 activated the MAPK signaling pathway by enhancing the transcription and stability of MAPK6 via recruiting TATA-box binding protein associated factor 15 in LUSC cells, resulting in the promotion of LUSC progression (86). Wang et al. discovered that lncRNA PCAT19 increased MAP2K4 levels by binding to miR-25-3p, thereby repressing the MAPK signaling pathway and LC progression (87). Moreover, our previous study discovered that circ-ZKSCAN1 increased the levels of FAM83A via sequestering miR-330-5p, leading to the inactivation of the MAPK signaling pathway and subsequent facilitation of NSCLC progression (88). In addition, Zhuang et al. revealed that circ-RAD23B increased MAP4K3 levels by sequestering miR-142-3p, thereby enhancing the activity of the MAPK signaling pathway and promoting NSCLC progression (89). Hu et al. discovered that circCNN2 activated the MAPK signaling pathway through upregulating E2F TF 1 via sponging miR-184, thereby promoting LUSC progression (90).

### Wnt/β-catenin pathway

The Wnt/β-catenin signaling pathway governs various physiological processes, such as embryo development and tissue homeostasis, and its aberrant activation is tightly linked with cancer progression (91). Some ncRNAs (e.g., miR-1275, miR-199, MIR4435-2HG, and FLVCR1-AS1) have been demonstrated to contribute to LC progression through the Wnt/β-catenin signaling pathway (92–97). Furthermore, multiple lncRNAs and circRNAs can regulate the Wnt/β-catenin signaling pathway by serving as competing endogenous RNA (ceRNA) for miRNAs in LC. Liu et al. showed that lncRNA RP11-79H23.3 knockdown enhanced the activity of the Wnt/β-catenin signaling pathway in NSCLC cells by sequestering miR-29c, resulting in the facilitation of LC progression (98). Yang et al. revealed that circ_0017109 knockdown decreased FZD4 levels by releasing miR-671-5p, resulting in the enhancement of the Wnt/β-catenin signaling pathway and subsequent facilitation of NSCLC progression (99). In addition, circCDR1 was found to activate the Wnt/β-catenin signaling pathway by interacting with SRSF1, thereby facilitating PM2.5-induced LC development (100).

### Notch pathway

The Notch signaling pathway is involved in the regulation of multiple biological processes, such as cell fate determination, embryo formation, and organism homeostasis (101). The dysregulation of the Notch signaling pathway contribute to many aspects of LC progression, including uncontrolled proliferation, cancer cell stemness, and TME (102). Ji et al. found that miR-34a inactivated the Notch signaling pathway by downregulating Hes-1, Notch-1, and Survivin, resulting in the suppression of cell growth and invasiveness and facilitation of apoptosis in NSCLC cells (103). Xue et al. showed that miR-200 modulated the crosstalk of LUAD cells with adjacent cancer-associated fibroblasts (CAFs) by targeting Jagged1 and Jagged2 (Notch ligands), thereby activating the Notch signaling pathway in CAFs and subsequently repressing LUAD metastasis (104). Furthermore, exosomal AGAP2-AS1 activated the Notch signaling pathway in LC cells by upregulating Notch2 via sequestering miR-296, leading to the enhancement of cell malignant behaviors (105). SNHG11 upregulated Notch3 by sponging miR-193a-5p, thereby activating the Notch signaling pathway and subsequently facilitating LUAD progression (106). In addition, circ_0000190 was found to counteract the repression of luteolin on LC progression by activating the Notch-1 signaling pathway via sponging miR-130a-3p (107).

### Other pathways

NcRNAs can also play a role in regulating LC progression through other signaling pathways. Multiple ncRNAs (e.g., miR-520a-3p, lncRNA MIR503HG, and circ_cMras) have been shown to suppress LC development by inactivating the NF-κB pathway, whereas some other ncRNAs (e.g., miR-135b and lncRNA SNHG5) can promote LC progression by enhancing the NF-κB pathway (108–112). Furthermore, lncRNA H19 enhanced the STAT3 signaling pathway by increasing STAT3 levels via sequestering miR-17, thereby promoting the progression of NSCLC (113). Hsa_circ_0002874 repressed the p53 signaling pathway by upregulating MDM2 (the E3 ubiquitin ligase of p53) via sponging miR-1273f, thereby enhancing the PTX resistance of NSCLC cells (114). In addition, hsa_circ_0001946 suppressed cisplatin resistance in NSCLC cells by modulating the NER signaling pathway (115).

## NcRNA in LC proliferation and apoptosis

Uncontrolled proliferation and escape from apoptosis are the most defining characteristics of tumor cells. However, the regulatory network involved in proliferation and apoptosis remains unclear and need to be further clarified (116). NcRNAs has been demonstrated to be vital regulators of the two cellular processes in LC. Luo et al. discovered that the levels miRNA-144-5p were significantly increased in LUAD, and its upregulation suppressed proliferation and promoted apoptosis in LUAD cells by targeting CDCA3 (117). Han et al. showed that miR-4491 was remarkably upregulated in NSCLC cells. Its overexpression facilitated proliferation and repressed apoptosis in NCI-H1650 cells through targeting TRIM7 (118). LncRNAs and circRNAs can also modulate LC proliferation and apoptosis via serving as ceRNAs for miRNA. For example, lncRNA-UCA1 was found to upregulate VEGF-A via sequestering miR-383, thereby facilitating proliferation and inhibiting apoptosis in HCC-78 cells (119). Circ_0000520 increased the levels of breast cancer-overexpressed gene 1 via sequestering miR-512-5p, resulting in the facilitation of proliferation and induction of apoptosis in LC cells (120).

Some cell cycle-related proteins are identified as downstream targets of ncRNAs (121), indicating that ncRNAs may participate in the modulation of the proliferation and apoptosis in LC through influencing cell cycle process. For instance, Huang et al. discovered that let-7c-5p overexpression arrested cells in G0/G1 phase by targeting cell division cycle 25A, resulting in the repression of proliferation and the promotion of apoptosis in LUAD cells (122). Wang et al. revealed that lnc-TMEM132D-AS1 induced M2/G-phase cell cycle arrest, facilitated proliferation, and repressed apoptosis in NSCLC cells by upregulating CD39 via sponging miR-766-5p (123). In addition, circPIM3 was found to increase TNFAIP8 levels by sponging miR-338-3p, thereby repressing apoptosis and promoting cell cycle progress and proliferation in taxol-resistant A549 and PC9 cells (124). Taken together, these findings strongly suggest that ncRNAs play pleiotropic roles in LC progression.

## NcRNA in LC invasion and metastasis

The invasion and metastasis of tumor cells are major causes of cancer recurrence and mortality (125, 126). Therefore, elucidating the potential mechanisms involved in invasion and metastasis is essential for developing therapeutic strategies to ameliorate prognosis for LC patients. NcRNAs have been proven to serve as key modulators that mediate invasion and metastasis in LC. For example, miR-96-5p was found to activate the MAPK signaling pathway by targeting domain-binding protein 2, leading to the repression of invasion and metastasis in LC cells (127). MiR-520a-3p decreased NF-κB p65 levels by targeting AKT1, thereby inactivating the NF-κB pathway and ultimately suppressing cell invasion and metastasis in NSCLC cells (128). Moreover, lncRNA TEX41 suppressed the PI3K/AKT signaling pathway by increasing Runx2 expression, leading to the facilitation of invasion, metastasis, and autophagy in LUAD cells (129). Circ_0000376 silencing decreased PDPK1 expression by releasing miR-545-3p, thereby repressing invasion and metastasis in NSCLC cells (130).

EMT is a biological process of cellular morphological alterations in which epithelial cells obtain mesenchymal characteristics. Recent studies suggest that ncRNA dysregulation endows LC cells with invasive and metastatic characteristics by altering EMT (84, 131, 132). Exosomal miR-181b-5p and let-7c-5p was found to inhibit the EMT process in BEAS-2B cells by modulating the MAPK signaling pathway, resulting in the enhancement of migratory and invasive ability in BEAS-2B cells (84). Moreover, Yang et al. showed that lncRNA PCAT6 significantly repressed the migration, invasion, and EMT of A549 and H1975 cells by increasing EGFR expression via sequestering miR-545-3p (131). Liu et al. revealed that circSCN8A suppressed invasion, metastasis, and EMT in NSCLC cell lines. Mechanistically, circSCN8A increased the levels of ACSL4 by sponging miR-1290, thereby repressing NSCLC progression (132). Collectively, as the key modulators of invasion and metastasis during LC progression, ncRNAs have presented great value as target candidates in LC treatment.

## NcRNA in LC angiogenesis

Angiogenesis denotes the development of new vessels from existing ones, by which tumor cells acquire sufficient material supplement for their growth (133). Targeting angiogenesis is considered a promising strategy in cancer treatment. NcRNAs are modulators of angiogenesis in LC. Gan et al. found that let-7d-5p expression was remarkably increased in LC cells treated with Trametes robiniophila, and its upregulation inhibited angiogenesis and tumor growth in LC by targeting NAP1L1 (134). Chang et al. demonstrated that exosomal miR-197-3p from LUAD cells could facilitate the angiogenesis of HUVECs by directly downregulating TIMP2/3 (135). Furthermore, Pan et al. discovered that LANCL1-AS1 upregulation dramatically repressed the angiogenesis of NSCLC cells by upregulating glia maturation factor gamma via sponging miR-3680-3p (136). Wang et al. revealed that ZNRD1-AS1 upregulation increased tensin 1 levels by sponging miR-942, thereby suppressing LC angiogenesis (137). In addition, circ_0043256 upregulation remarkably repressed angiogenesis in LC cells by upregulating KLF2 via absorbing miR-1206 (138). In-depth investigations are needed to further clarify the ncRNA action in angiogenesis, which may bring significant advantage for the development of theoretical basis in LC treatment.

## NcRNA in LC tumor microenvironment

Tumor microenvironment (TME) is a highly complicated ecosystem that contains tumor cells, nontumoral cells, and various cytokines and chemokines generated by them. The continuous interaction between tumor cells and TME contributes to carcinogenesis, metastasis, and drug resistance (139). NcRNAs are involved in LC development through targeting the cellular components of TME, such as CAFs and tumor-associated macrophages (TAMs) (140–145). Liu et al. discovered that CAF-derived exosomal miR-200 inhibited morphological and metastatic characteristics of NSCLC cells by downregulating ZEB1 (140). Enukashvily et al. showed that satellite lncRNA knockdown reduced cell aging and attenuated inflammatory CAF phenotype in human lung fibroblasts (141). Furthermore, Li et al. revealed that LINC01798 remarkably increased ITGA8 levels through absorbing miR-17-5p, resulting in the alteration of TME and stemness in LUAD cells (142). As the main immune cell population in the TME, TAMs play vital roles in shaping the TME (143). NSCLC cell-derived exosomal miR-181b was found to enhance TAM M2 polarization through the activation of the STAT3 signaling pathway (144). Moreover, Wu et al. demonstrated that LINC01094 activated the transcription of CCL7 by facilitating the shuttling of SPI1 from cytoplasm to nucleus, resulting in the accumulation of M2 TAMs and the dissemination of LUAD cells (145). In addition, exosomal circFARSA was found to polarize TAMs to an M2 phenotype by enhancing the activation of the PI3K/AKT pathway. NSCLC cells co-cultured with TAMs transfected with circFARSA exhibited enhanced EMT and metastasis (146).

## NcRNA in LC tumor stemness

Cancer stem cell (CSC) belongs to a specific type of self-renewal cells, which is considered the major factor contributing to metastasis, chemoresistance, and recurrence in cancer (147). Elucidating the detailed mechanism involved in the modulation of CSC functions may bring significant benefit to the development of individualized treatment of LC patients. NcRNAs are key regulators of stemness in LC cells (148–151). Moro et al. discovered that miR-486-5p facilitated apoptosis and decreased viability in CD133+ lung CSCs by inactivating the PI3K/AKT pathway, leading to the inhibition of the tumor-initiating roles of these cells (148). Liu et al. demonstrated that miR-1246 knockdown attenuated the stemness of LC cells by directly targeting TRIM17 (149). Furthermore, the overexpression of ADAMTS9-AS1 was found to significantly increase NPNT expression by sequestering miR-5009-3p, thereby repressing the stemness of LUDA-CSCs (150). Lu et al. revealed that TDRG1 was remarkably increased in lung CSCs compared with parental LC cells. TDRG1 overexpression enhanced the stemness of lung CSCs by upregulating Sox2 (stemness marker) via binding to its mRNA (151). In addition, circRACGAP1 enhanced stemness and metastasis in NSCLC cells via promoting SIRT3-mediated RIF1 deacetylation (152). Collectively, these studies suggest that ncRNAs are key modulators of stemness in LC cells. However, their regulatory mechanisms remain not fully understood, which need to be further elucidated.

## NcRNAs in LC chemoresistance

Chemotherapy is a well-established treatment method for distinct cancer types and can significantly extend patients’ life spans, but the development of chemoresistance limits its further utilization and ultimately results in patients’ death (153). NcRNA dysregulation is closely correlated with the emergency of chemoresistance in LC treatment (154). Our previous study showed that miR-608 was remarkably downregulated in NSCLC samples. MiR-608 overexpression in NSCLC cells facilitated doxorubicin-induced apoptosis by targeting TFAP4 (155). Vinciguerra et al. discovered that miR-301a was dramatically decreased in cisplatin-resistant NSCLC cells. The overexpression of miR-301a downregulated GLIPR1 by targeting Fra-2, thereby improving cisplatin resistance in NSCLC cells (156). Besides, miR-936 was significantly downregulated in NSCLC cells, and its overexpression inactivated the Galphaq Rho GTPase pathway by targeting GPR78, resulting in the repression of cisplatin resistance in NSCLC cells (157). Furthermore, Yu et al. demonstrated that lncRNA LOC85009 inhibited ATG5-induced autophagy by decreasing the stability of upstream TF 1 via sequestering ubiquitin-specific proteinase 5, thereby triggering cell apoptosis and suppressing docetaxel resistance in LUAD cells. Interestingly, exosomal LOC85009 derived from LUAD cells enhanced docetaxel sensitivity in docetaxel-resistant cells (158). Liu et al. discovered that DDX11-AS1A was remarkably increased in LUAD, and its upregulation attenuated paclitaxel sensitivity in LUAD cells through promoting DNA damage repair (159). In addition, CircPIM3 decreased taxol sensitivity and inhibit apoptosis in taxol-resistant NSCLC cells. Mechanistically, circPIM3 upregulated tumor necrosis factor-alpha-induced protein-8 via absorbing miR-338-3p, thereby enhancing taxol resistance in NSCLC cells (124).

## NcRNA and tobacco smoking in LC

Tobacco smoking is considered the major risk factor of LC that generates long-lasting and progressive impairment to the lung tissue (160). Some ingredients in tobacco have been shown to contribute to tumorigenesis and progression of LC by promoting malignant behaviors of cancer cells and inducing chronic inflammation (161, 162). However, the exact mechanisms of tobacco smoking in LC remain largely unknown. Recent studies suggest that ncRNAs play vital roles in pathogenesis of tobacco smoking-induced LC (163–165). Tobacco-specific nitrosamine 4-(methylnitrosamino)-1-(3-pyridyl)-1-butanone (NNK) is a well-studied strong carcinogen. Kalscheuer et al. discovered that the levels of miR-101, miR-126*, miR-199 and miR-34 were significantly downregulated in male rats treated with NNK, indicating the potential value of these miRNAs as diagnostic biomarker for early LC development. Functional analysis revealed that NNK exerted its oncogenic role by increasing cytochrome P450 (CYP) 2A3 levels via downregulating miR-126* (163). Chen et al. showed that NNK treatment decreased lncRNA AC007255.8 expression by inducing its promoter hypermethylation, resulting in the promotion of proliferation and the suppression of apoptosis in human bronchial epithelial Beas-2B cells (164). Furthermore, Hua et al. demonstrated that circ_0035266 regulated the inflammatory responses of Beas-2B cells to NNK and lipopolysaccharide (LPS) by altering the secretion of IL-6 and IL-8. Mechanistically, circ_0035266 overexpression upregulated DDX3X by sponging miR-181d-5p, thereby facilitating IL-6 and IL-8 secretion and ultimately resulting in the enhancement of inflammatory responses of cells to NNK and LPS (165). They also found that circ_0035266 knockdown significantly repressed the proliferation, cell cycle process, and migration of Beas-2B cells treated with NNK and LPS (166). Nicotine is a primary alkaloid derived from tobacco plants. Liu et al. revealed that the levels of miR-218 were remarkably decreased in NSCLC cells treated with nicotine and its downregulation facilitated the expression of CDK6, leading to the promotion of cell proliferation (167). Zhao et al. showed that the nicotine-induced upregulation of LINC00460 promoted the proliferation and migration of NSCLC cells and the inhibition of cell apoptosis (161). In addition, Zong et al. demonstrated that lncRNA CCAT1 was significantly upregulated in human bronchial epithelial (HBE) cells treated with cigarette smoke extract (CSE). CCAT1 overexpression activated the ERK signaling pathway by sponging miR-152-3p, resulting in the enhancement of inflammation in CSE-treated HBE cells (168). Collectively, these findings strongly suggest that ncRNAs are key regulators in tobacco smoking-induced LC progression. Understanding their exact mechanisms in tobacco smoking -associated LC may provide novel insights in the development of individualized treatment of tobacco-using patients with LC.

**Figure 2:**
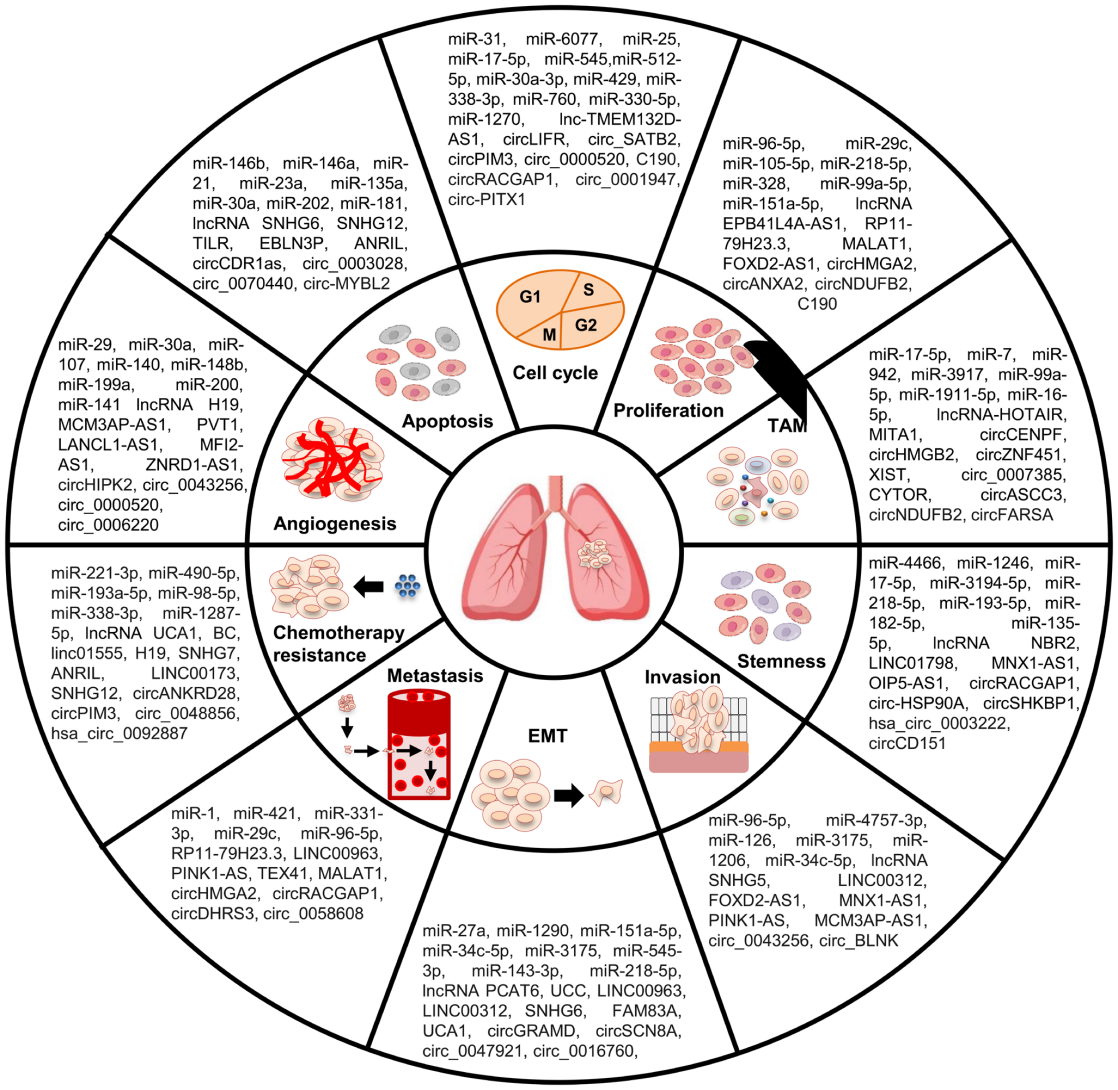
Role of ncRNAs in malignant behaviors of LC cells. NcRNAs participate in the regulation of LC malignant behaviors, including cell apoptosis, proliferation, cell cycle, invasion, metastasis, EMT, TAM, stemness, angiogenesis, and chemoresistance.

In summary, ncRNAs can play pleiotropic roles in almost all aspects of LC occurrence and development, such as EMT, apoptosis, angiogenesis, TME, stemness, and chemoresistance (**Figure 2**). The key functions of ncRNAs in the development of LC malignant characteristics endow them with great clinical application value in LC treatment.

## Clinical implications of ncRNA in LC

### NcRNA in LC diagnosis and prognosis

Currently, most LC patients have poor clinical outcomes because of the lack of effective early diagnosis and prognostic assessment means (33). Multiple proteins (e.g., PSA, CA-125, and CYFRA 21-1) have been applied in LC treatment by serving as biomarkers. However the unsatisfactory accuracy and reliability restrict their further utilization (169). NcRNAs have exhibited differently expressed patterns, high stability and specificity, and detectability (170–172). These specific features endow them with great value as noninvasive biomarkers for LC patients (**Tables 1**, **2**). Wang et al. performed an in-depth meta-analysis and discovered that the area under the curve (AUC) value for miR-21 in distinguishing LC was 0.87, with 77% sensitivity and 86% specificity. Moreover, high miR-21 levels were remarkably associated with overall survival (OS) in LC patient (170). Wu et al., found that miR-340 was dramatically downregulated in plasma from NSCLC patients, whereas miR-450b-5p were upregulated. The AUC values for circulating miR-340 and miR-450b-5p in distinguishing NSCLC were 0.740 and 0.808, respectively. Furthermore, lower miR-340 and higher miR-450b-5p were significantly correlated with prognosis in NSCLC (171).

**Table 1:** NcRNAs as diagnostic or prognostic biomarkers in LC.

LC subtypes	NcRNA subtypes		Samples	Techniques	Expression patterns	Biomarker types	Potential values	Reference
NSCLC	miRNA	miR-21-5p	Blood mononuclear cells	RT-qPCR	Up	Diagnosis	100% sensitivity and 55.3% specificity.	(173)
		miR-30	Serum	RT-qPCR	Down	Diagnosis Prognosis	AUC = 0.802, 76.0% sensitivity and 75.9% specificity. The OS of miR-30 low expression patients was shorter than high expression patients (p < 0.05).	(174)
		miR-7-5p	A549, H358, H520, and SPC-A1	qPCR	Down	Prognosis	Low miR-7-5p expression was closely associated with poor prognosis of NSCLC patients (p = 0.014).	(175)
		miR-155, miR-222	Serum	qPCR	Up	Diagnosis	High levels of miR-155 and miR-222 are closely correlated with worse prognosis in NSCLC patients (p = 0.014).	(176)
		miR-184	Serum exosomes	qPCR	Up	Diagnosis Prognosis	AUC = 0.927, 87.61% sensitivity and 84.02% specificity. The levels of miR-184 in serum exosomes is significantly correlated with prognosis in NSCLC (p < 0.05).	(13)
		miR-223	Serum	ddPCR	Up	Diagnosis	AUC = 0.753.	(177)
		miR-339-3p	Serum	RT-qPCR	Up	Diagnosis	AUC = 0.616.	(178)
		miR-21, miR-23a	Plasma	qRT-PCR	Up	Prognosis	The expression of miRNA-21 and miRNA-23a was higher in plasma from NSCLC patients with distant metastasis compared with patients without metastasis (p < 0.0001).	(179)
		miR-629	Serum	RT-qPCR	Up	Prognosis	High serum miR-629 NSCLC patients suffered poorer OS and DFS than those in the low serum miR-629 patients.	(180)
		miR-92a	Tissue	PT-PCR	Up	Prognosis	MiR-92a levels were significantly correlated with prognosis in NSCLC (p = 0.036).	(181)
	lncRNA	TA73-AS1, CRNDE	Plasma	qRT-PCR	Up	Diagnosis	AUC for TA73‐AS1 was 0.822; AUG for CRNDE was 0.815; High expression of the two plasma lncRNAs are associated with worse TFS in NSCLC patients.	(172)
		SLC9A3-AS1	Serum	RT-qPCR	Up	Diagnosis Prognosis	AUC = 0.74. High SLC9A3-AS1 levels were correlated with shorter OS (p = 0.033) and RFS (p = 0.031).	(182)
		HOTAIR	Tissue	FISH	Up	Diagnosis	AUC = 0.801, 52.3% sensitivity and 86.9% specificity.	(183)
		RP5-977B1	Serum exosomes	qRT-PCR	Up	Diagnosis Prognosis	AUC = 0.8899. High RP5-977B1 levels were significantly correlated with poor prognosis in NSCLC (p = 0.036).	(184)
		ELFN1-AS1	Tissue	RT-qPCR	Up	Prognosis	High ELFN1-AS1 levels were significantly correlated with OS in NSCLC (p = 0.021).	(185)
	circRNA	circFOXP1	Serum	qRT-PCR	Up	Diagnosis	AUC = 0.88.	(186)
		hsa_circ_0069313	Serum exosomes	qRT-PCR	Down	Diagnosis	AUC = 0.749.	(187)
		hsa_circ_0023179	Serum	qRT-PCR	Up	Diagnosis	AUC = 0.831, 77% sensitivity and 86% specificity.	(188)
		hsa_circ_0070354	Serum	qRT-PCR	Up	Diagnosis Prognosis	AUC = 0.660, 52.63% sensitivity and 76.29% specificity. High hsa_circ_0070354 levels in NSCLC were significantly correlated with poor prognosis (p < 0.001).	(189)
		circRNA_001846	Serum	qRT-PCR	Up	Diagnosis	AUC = 0.872, 78.2% sensitivity and 81.1% specificity.	(190)
SCLC	miRNA	miR-92a-2	Plasma	qRT-PCR	Up	Diagnosis	AUC = 0.761, 56% sensitivity and 100% specificity.	(191)
		miR-375, miR-92b	Plasma	qRT-PCR	Up	Diagnosis Prognosis	AUC for miR-375 was 0.766; AUC for miR-92b was 0.791; The two miRNAs were closely correlated with reduced progression-free survival in SCLC.	(192)
		miR-92a-2*	Tissue	qRT-PCR	Up	Prognosis	Higher miR-92a-2* levels were correlated with poor survival in SCLC.	(193)
	lncRNA	KCNQ1OT1	SCLC cell lines H69AR and H69	RT-qPCR	Up	Prognosis	High KCNQ1OT1 levels were significantly correlated with poor prognosis in SCLC.	(194)
		CCAT2	Tissue	qRT-PCR	Up	Prognosis	High level of CCAT2 was associated with short OS of SCLC patients (p = 0.007).	(195)
		AK09398	Tissue	qRT-PCR	Up	Prognosis	High level of AK09398 was associated with poor OS and PFS in SCLC (p < 0.001).	(196)
	circRNA	cESRP1	SCLC cell	qRT-PCR and FISH	Down	Prognosis	cESRP1 expression was associated with OS in SCLC patients (p = 0.0017).	(197)
		FECRs	Serum exosome	qRT-PCR and FISH	Up	Prognosis	Exosomal FECR1 levels were closely correlated with shorter survival in SCLC (p = 0.038).	(198)

**Table 2:** NcRNAs as diagnostic or prognostic biomarkers in different types of NSCLC.

NSCLC subtypes	NcRNA subtypes		Samples	Techniques	Expression patterns	Biomarker types	Potential values	Reference
LUAD	miRNA	miR-30d-5p	Serum	RT-qPCR	Down	Prognosis	High of miR-30d-5p was closely correlated with longer RFS in LUAD (p = 0.02).	(199)
		miRNA-30a-5p	Serum	qRT-PCR	Down	Prognosis	AUC = 0.902. Low levels of miR-30d-5p were closely correlated with worse clinical outcomes in LUAD.	(200)
		miR-125b-5p	Tissue	Bioinformatics	Down	Prognosis	AUC = 0.768. Low levels of miR-125b-5p were closely associated with poor OS and DFS in LUAD (p < 0.0001).	(201)
		miR4732-5p, miR451a, miR486-5p, and miR139-3p	Serum exosome	qRT-PCR	UP	Diagnosis	AUC = 0.8554, 91.07% sensitivity and 66.36% specificity.	(19)
	lncRNA	CASC11	Tissue, plasma	qRT-PCR	UP	Diagnosis Prognosis	CASC11 exhibited diagnostic value in LUAD (p < 0.0001). High CASC11 levels were closely correlated with poor prognosis in LUAD (p < 0.05).	(202)
		IPW	Tissue, cell	Bioinformatics, qRT-PCR	Down	Prognosis	High levels of miR-370 were closely correlated with shorter OS in LUAD (p = 0.045).	(203)
		SIGLEC17P	Tissue	Bioinformatics, qRT-PCR	Down	Prognosis	AUC = 1.000 (p < 0.01). LUAD patients with high SIGLEC17P levels exhibited good OS (p = 0.0009) and RFS (p = 0.0053).	(204)
	circRNA	hsa_circ_101555, hsa_circ_008068	Tissue, plasma	qPCR	Up	Diagnosis	AUC for hsa_circ_101555 was 0.708 (76.67% sensitivity and 60.00% specificity); AUC for hsa_circ_008068 was 0.624 (63.33% sensitivity and 53.33% specificity).	(205)
		hsa_circ_0001492, hsa_circ_0001439, hsa_circ_0000896	Serum, serum exosome	qRT-PCR	Up	Diagnosis	The AUC value of the combination of exosomal hsa_circ_0001492, hsa_circ_0001439, and hsa_circ_0000896 was 0.805.	(206)
LUSC	miRNA	miRNA-126-3p	Tissue	qRT-PCR	Down	Diagnosis Prognosis	AUC = 0.6748 (p = 0.018). LUSC patients with low miRNA-126-3p levels exhibited poor OS (p = 0.0004).	(207)
		miR-486-5p	Tissue, ell	Bioinformatics, qRT-PCR	Down	Diagnosis	AUC = 0.9082 (95% CI: 3.47-1.03; p = 0.0003).	(208)
		miR-1	Tissue	Bioinformatics	Down	Diagnosis	AUC = 0.9096, with 71% sensitivity and 88% specificity.	(209)
	lncRNA	TTTY16, POU6F2-AS2, CACNA2D3-AS1	Tissue	Bioinformatics	Up	Prognosis	The AUC value for the three lncRNAs correlated with 3-year survival was 0.629 in LUSC patients.	(210)
		LINC02323	Tissue	Bioinformatics	Up	Prognosis	LUSC patients with high LINC02323 levels exhibited poor OS (p = 0.0089).	(211)
	circRNA	hsa_circ_0014235, hsa_circ_0025580		qRT-PCR	Up	Diagnosis	The AUC values of hsa_circ_0014235 and hsa_circ_0025580 were 0.8254 and 0.8003 in LUSC patients, respectively.	(212)

LncRNAs and circRNAs have also been utilized in LC clinical research. Yuan et al. discovered that the plasma levels of CRNDE and TA73-AS1 were significantly increased in NSCLC tissues. Their AUC values in distinguishing NSCLC were 0.822 and 0.815, separately. Moreover, their plasma levels were also closely correlated with poor tumor-free survival in NSCLC (172). Zhang et al. showed that NPSR1-AS1 was much higher in LUAD samples compared with benign samples. The AUC value for high NPSR1-AS1 in the diagnosis of LUAD was 0.904, with a 95% CI ranging from 0.881 to 0.927. Furthermore, the levels of NPSR1-AS1 exhibited positive correlation with OS in LUAD patients (213). Zou et al. demosntrated that AUC for serum circERBB2 in distinguishing NSCLC was 0.871, which was higher than CYFRA21-1 (0.693) and CEA (0.861). Moreover, LC patients with low circERBB2 levels had higher 36-month cumulative survival rate than patients with high circERBB2 levels (p < 0.05) (214). In addition, Li et al. showed that high levels of hsa_circ_001010 and hsa_circ-ZNF609 was negatively correlated with OS and DFS, whereas low levels of hsa_circ-CRIMI1, hsa_circ-EPB41L2, and hsa_circ_0072309 was positively associated with OS and DFS in LUAD. The four circRNAs also exhibits great potential in distinguishing LUAD (215).

### NcRNA in LC treatment

As the key regulators of LC progression, ncRNAs have displayed huge therapeutic potential (133). Targeting oncogenic ncRNAs represents a highly feasible solution for patients to improve LC interventions. Chu et al. showed that miR-96-5p levels were dramatically increased in LC tissues, and its upregulation altered the expression of Bax, MMP9, and Bcl-2 through downregulating domain-binding protein 2, thereby facilitating invasion and proliferation in H1299 cell lines (127). In another study by Lv et al., lncRNA MNX1-AS1 was remarkably increased in LC samples, and its downregulation repressed the proliferation, migration, invasion, and sphere-forming abilities of LC CSCs by activating myosin IG (216). Furthermore, Sun et al. revealed that circ_0000376 knockdown decreased PDPK1 levels by releasing miR-545-3p, thereby suppressing NSCLC progression (130). Upregulating tumor-suppressive ncRNAs in cancer cells could be another effective strategy in LC treatment. For example, miR-1 overexpression repressed cell growth and oncogenic signaling in SCLC cells by targeting CXCR4. Consistent with this, intracardiac injection of miR-1 SCLC cells in mice exhibited a reduction in distant tissue metastasis (217). Furthermore, Gao et al. demonstrated that lncRNA FAM138B suppressed cell proliferation and invasion by targeting miR-105-5p in NSCLC cells (218). Song et al. revealed that circANKRD28 overexpression enhanced cisplatin sensitivity in NSCLC cells through increasing SOCS3 levels via absorbing miR-221-3p (219). To summarize, therapeutic strategies that directly target ncRNAs or use ncRNAs will bring significant benefit to the development of individualized treatment of LC patients.

## Conclusion

**Figure 3:**
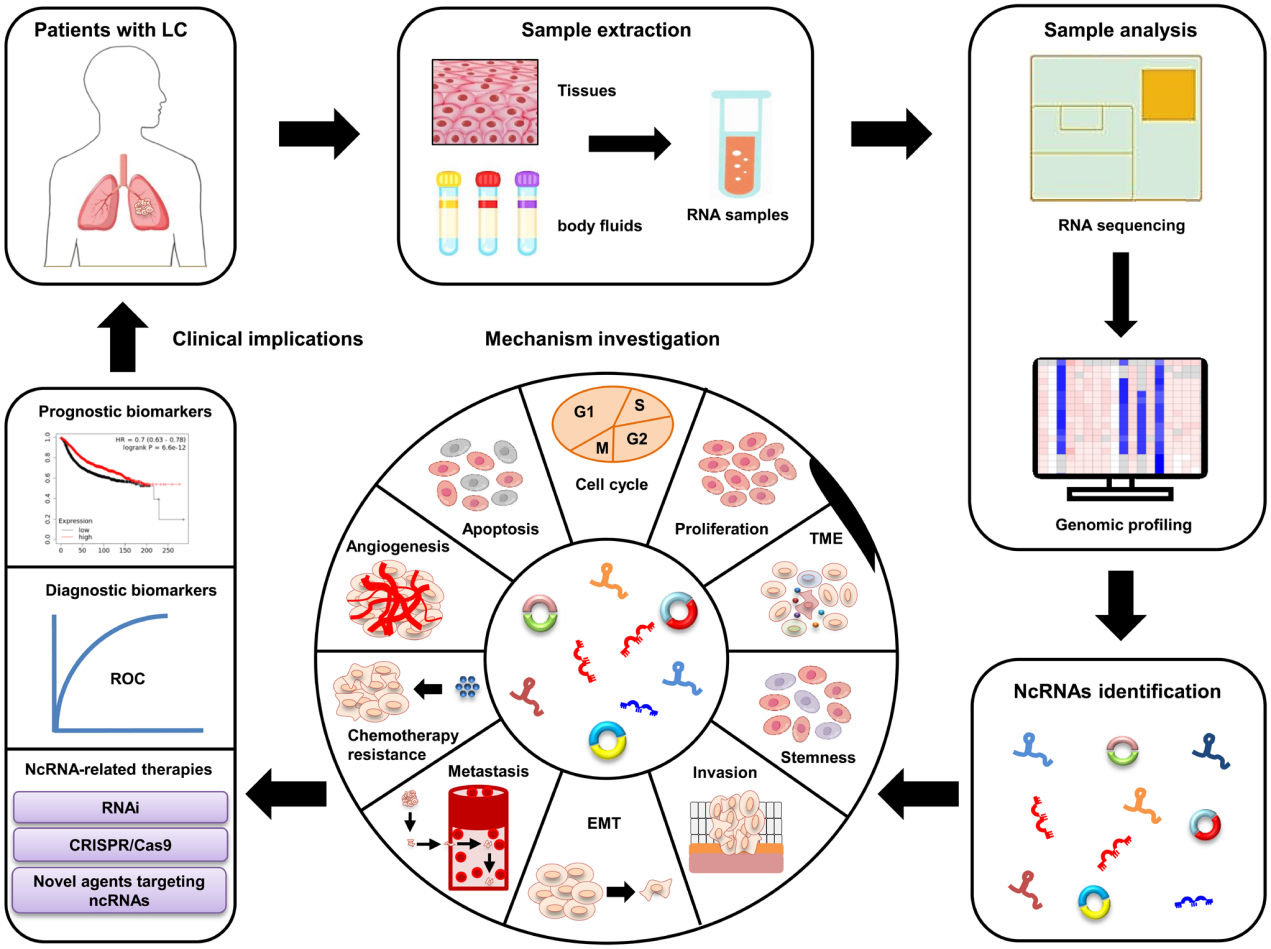
Clinical applications of ncRNAs in LC. The dysregulated ncRNAs are identified from LC patient samples using RNA sequencing and bioinformatics. *In vitro* and *in vivo* studies are performed to further clarify the underlying mechanisms of these ncRNAs involved in LC progression. Large patient cohorts are used to validate their potential as diagnostic and prognostic biomarkers. Novel ncRNA-based therapeutic strategies are developed for LC patients.

LC, the most commonly diagnosed type of cancer in respiratory system, severely shortens patients’ life expectancy. The pathogenesis of LC is very complex and still unclear. Clarifying the regulatory mechanisms involved in LC progression is extremely urgent for developing efficient therapies of LC patients. Recent studies have identified a large amount of differently expressed ncRNAs (e.g., miRNAs, lncRNAs, and circRNAs) in LC. These ncRNAs play vital roles in LC progression by influencing almost all biological processes, such as cell invasion, autophagy, CSCs, and chemoresistance (28–30). Moreover, the differently expressed ncRNAs are easily examined in body fluids (e.g., serum and lymph) of LC patients, and their differentiated expression patterns are also closely correlated with some pathological characteristics, including tumor-free survival, OS, and DFS (172, 215). These unique characteristics mean that ncRNAs are valuable candidates of non-invasive biomarker and target in LC treatment (**Figure 3**). However, some challenges (e.g., ununified standardization strategies, unknown side effects, and insufficient patient size) still exist, which should be addressed before applying ncRNAs in LC clinical treatment. Nevertheless, recent studies strongly suggest that ncRNAs are effective biomarkers and promising targets for LC patients. Future investigations should focus on elucidating the exact functions of ncRNA in LC pathogenesis and developing novel ncRNA-based therapeutic strategies.

## Author contributions

YL: Original draft preparation, writing—review and editing. WD: Data curation. JW: Data curation. XA: Original draft preparation, funding acquisition. JX: Writing—conceptualization, original draft preparation; writing—review and editing. All authors contributed to the article and approved the submitted version.
